# Original Translations

**Published:** 1880-11

**Authors:** 


### Original Translations.

MENSTRUATION AFTER A REMOVAL OF THE UTERUS.—Dr. Tillaux reported to the Academie de Médecine, Paris, August 31st, the case of a woman in whom he had removed the body of the uterus sixteen months previously. Menstruation returned more frequent and in smaller quantity from the remaining portion. Another woman continued to menstruate after a removal of the ovaries.

INFLUENCE OF OXYGENATED WATER ON FERMENTS.—Dr. Regnard has mixed a few drops of oxygenated water with yeast, urine, milk, wine, white of egg, etc., and after a month none of these had undergone fermentation.—*Société de Biologie.*

PARIS.—Dr. Bouchardat writes to the Academie de Médecine that the most frequent cause of infantile mortality in that city is diarrhœa ; that the latter is mostly caused or induced by the use of milk which is taken twenty-four hours after milking, when the lactic fermentation has already begun. The best treatment is to resume nursing from the breast.—Dr. M. J. Guérin reports to the same body, Sept. 7, favorable results in the treatment of that disease from the addition of pulverized charcoal to the milk.

RESULTS OF THE TREATMENT OF AORTIC ANEURISMS BY GALVANO-PUNCTURE.—Dr. L. H. Petit reports 114 cases treated. The interrupted current was employed in 111 cases. Of the 114, sixty improved, thirty-eight died without improvement, three were treated without result, and four doubtful ; thirty-eight died within a year, after a marked improvement; ten after one to two years. The rest survived from two to five years. The rupture of the aneurismal sac was constated forty times in the fatal cases which remained under observation.

DR. GENTILHOMME, of Rheims, refers acne to the presence of a parasite *demodex* in the sebaceous glands. The destruction of this parasite requires a long and tedious treatment, and the disease is likely to return if a few parasites have escaped.

PROF. TOUSSAINT relates more anthrax vaccinations. Some of the animals died, perhaps because some germs had remained in the serum used after filtering. Animals once vaccinated are not susceptible to a second vaccination.—*Revue Medicale*, Sept. 4th.

RECIPROCAL INFLUENCE OF PREGNANCY AND HEART DISEASE. By Dr. Ch. Parak. In a large number of recorded cases, patients remained stationary in 25 per cent., grew worse in 65.74 per cent., died in 38 per cent., and improved in 26 per cent. Labors at term, 58.87 per cent. ; before term, 41 per cent., including abortions.

LARYNGOLOGICAL CONGRESS AT MILAN, SEPT. 2D.—Dr. Maurice Schmidt has observed nineteen cases of phthisical laryngitis cured in 315 cases which he treated in the space of three years. Antiseptics preferred. Inhalations of Peruvian balsam, phenic acid, or glycerite of creosote. Local applications of a solution of nitrate of silver, 24 to 48 grs. to the ounce.—Dr. Caselli presents the case of a girl, nineteen years old, in whom he has removed the larynx, the pharyngo-buccal wall, the uvula and part of soft palate, the tonsils and part of base of the tongue. The introduction into the trachea of two canulas enables the patient to eat, and the use of a canula with a sort of musical arrangement enables her to speak.

PARIS, Aug. 17th.—A case of hydrophobia was treated by electricity—ten cells (Trouvé-Callaud) being used. The application of the electrodes to the larynx reduced the spasms every time. Patient died from a dispute with a medical attendant, which brought on coma.

NECROLOGY.—Dr. Delpech, a member of the Academie de Médecine, died Sept. 5th, aged 62, from an attack of angina pectoris.—Dr. Lepeyrère, a noted writer in the medical press, has lately died at Boulogne-sur-Seine.

A MEDICAL COLLEGE has just been established at Algiers, after an edict of the president of France. No European is admitted. Course consists of four consecutive terms (of three months each), and the graduates will be called *sous-officier de sante de l'Algerie.*

---

KLEBS ON THE SPECIFIC AGENT OF TYPHOID FEVER.—Prof. Klebs, of Prague, believes that he has discovered the micro-organism which constitutes the specific agent of typhoid fever, and develops his views in a paper entitled "Der Ileotyphus eine Schistomy cose," published in the *Archiv. für Experimentale Pathologie*, t. 12, p. 231, 1880 (*British Medical Journal*). Prof. Klebs has for a long time, assisted by his pupils, been making researches in this direction. He writes that he has been able to find, at the necropsy of twenty-four persons carried off by dothinenteritis, microbes in various organs—in the intestinal mucous membrane, in the thickness of the cartilages of the larynx, in the pia mater, in the foci of lobular pneumonia, in the mesenteric ganglia, in the parenchymata of the liver, and generally diffused in the organs which showed the most decided lesions. These micro-organisms showed themselves in the forms of rods about eighty micrometers in length and 0.5 to 0.6 micrometers in thickness. They have been constantly observed in the bodies of dothinenteric patients since the attention of Prof. Klebs was drawn to the subject, and they are always absent from the organs, and specially the intestines, of subjects who have died from any other disease than typhoid.—*Louisville Med. News.*

THE serious illness of Prof. T. D. Fitch, of this city, still is the occasion of anxiety to his many friends here. We hope in our next issue to announce the fact of his safety.

